# *Eurosurveillance* annual theme 2025: *Eurosurveillance* opens submissions on vaccine-preventable diseases in humans — today's challenges and tomorrow's opportunities

**DOI:** 10.2807/1560-7917.ES.2024.29.40.031024mis

**Published:** 2024-10-03

**Authors:** Eurosurveillance editorial team

**Affiliations:** 1 European Centre for Disease Prevention and Control (ECDC), Stockholm, Sweden

Vaccination is the most cost-effective life-saving medical intervention, estimated to save at least 3.5–5 million human lives each year from diseases like diphtheria, tetanus, pertussis, influenza and measles [1,2]. In the control of emerging infectious diseases as in the COVID-19 pandemic and recently mpox, vaccines have been vital [3-7]. Currently, there are vaccines to prevent more than 20 life-threatening diseases, and some vaccines against more diseases were either recently launched such as that against respiratory syncytial virus (RSV) infection or are under development such as a vaccine against Crimean-Congo haemorrhagic fever [8-10]. Prevention of several zoonotic diseases in humans, like rabies, brucellosis or Q fever, can be achieved by including vaccination of domestic food-producing animals, companion animals and/or wild animals in the control measures applying a One Health approach [11-17].

New vaccine concepts and technologies can offer more effective immunisation and efficient and more adjustable vaccine production by for instance, protecting against novel pathogens or multiple variants of a single pathogen.

Despite the clear benefits of vaccination, there are challenges. Vaccine coverage varies among and within countries for various reasons such as poor access to vaccination or vaccine hesitancy. The World Health Organization (WHO) implemented the global immunisation agenda 2030 (https://www.who.int/teams/immunization-vaccines-and-biologicals/strategies/ia2030) in 2021 to encourage, inspire and align activities on vaccination and immunisation.

Improving vaccine acceptancy can be challenging. Concerns about vaccine safety can be linked to vaccine hesitancy, but safety concerns are only one of many factors that may drive hesitancy. Robust studies on vaccine safety, access to vaccine, efficacy and effectiveness are important in increasing vaccination coverage.

## For our 2025 annual theme, we are therefore encouraging article submissions relevant for Europe, that address, but are not limited to, topics such as

-impact of vaccinations on public health: benefits and challenges
-interventions and incentives to increase and motivate vaccinations
-preparedness for new and emerging diseases: vaccination capacity, new technologies
-social determinants of health and vaccination including access to vaccines
-spatiotemporal distribution of vaccine uptake
-activities building trust in vaccines including transparent communication about vaccines’ benefit and safety
-vaccination policies
-vaccination registries
-vaccine efficacy and effectiveness
-vaccine hesitancy and vaccine fatigue
-modelling of vaccination scenarios

If you would like to submit a paper or ask for more information, please consult our instructions for authors regarding article types or contact the editorial team at eurosurveillance@ecdc.europa.eu.
